# Children Born Preterm at the Turn of the Millennium Had Better Lung Function Than Children Born Similarly Preterm in the Early 1990s

**DOI:** 10.1371/journal.pone.0144243

**Published:** 2015-12-07

**Authors:** Maria Vollsæter, Kaia Skromme, Emma Satrell, Hege Clemm, Ola Røksund, Knut Øymar, Trond Markestad, Thomas Halvorsen

**Affiliations:** 1 Department of Clinical Science, University of Bergen, Bergen, Norway; 2 Department of Pediatrics, Haukeland University Hospital, Bergen, Norway; 3 Department of Occupational Therapy, Physiotherapy and Radiography, Bergen University College, Bergen, Norway; 4 Department of Pediatrics, Stavanger University Hospital, Stavanger, Norway; University Children's Hospital Basel, SWITZERLAND

## Abstract

**Objective:**

Compare respiratory health in children born extremely preterm (EP) or with extremely low birthweight (ELBW) nearly one decade apart, hypothesizing that better perinatal management has led to better outcome.

**Design:**

Fifty-seven (93%) of 61 eligible 11-year old children born in Western Norway in 1999–2000 with gestational age (GA) <28 weeks or birthweight <1000 gram (EP_1999–2000_) and matched term-controls were assessed with comprehensive lung function tests and standardized questionnaires. Outcome was compared with data obtained at 10 years of age from all (n = 35) subjects born at GA <29 weeks or birthweight <1001 gram within a part of the same region in 1991–92 (EP_1991–1992_) and their matched term-controls.

**Results:**

EP_1999–2000_ had significantly reduced forced expiratory flow in 1 second (FEV_1_), FEV_1_ to forced vital capacity (FEV_1_/FVC) and forced expiratory flow between 25–75% of FVC (FEF_25–75_), with z-scores respectively -0.34, -0.50 and -0.61 below those of the term-control group, and more bronchial hyperresponsiveness to methacholine (dose-response-slope 13.2 vs. 3.5; p<0.001), whereas other outcomes did not differ. Low birthweight z-scores, but not neonatal bronchopulmonary dysplasia (BPD) or low GA, predicted poor outcome. For children with neonatal BPD, important lung-function variables were better in EP_1999–2000_ compared to EP_1991–1992_. In regression models, improvements were related to more use of antenatal corticosteroids and surfactant treatment in the EP_1999–2000_.

**Conclusions:**

Small airway obstruction and bronchial hyperresponsiveness were still present in children born preterm in 1999–2000, but outcome was better than for children born similarly preterm in 1991–92, particularly after neonatal BPD. The findings suggest that better neonatal management not only improves survival, but also long-term pulmonary outcome.

## Introduction

Since the 1990s most infants born extremely preterm or with extremely low birthweight (hereafter referred to as EP-born) in high-income countries have survived to discharge [1, 2]. Birth at this stage of pregnancy implies that gas exchange must take place in fetal lungs, often leading to the syndrome of bronchopulmonary dysplasia (BPD) [3]. Life-long pulmonary prospects after EP birth and BPD are unknown, and concerns have been expressed for future functional deficits, such as early onset respiratory insufficiency [4, 5].

Neonatal pulmonary autopsy data suggest that EP birth may lead to severe bronchoalveolar abnormalities, but structural data from later life are scarce [6]. However, a range of functional abnormalities have repeatedly been described in EP-born survivors, such as airway obstruction, bronchial hyperresponsiveness, pulmonary hyperinflation and impaired gas diffusing capacity. Generally, those with neonatal BPD do worse. This scenario applies to EP-born adults from the early era of neonatal intensive care units (NICUs) as well as to children exposed to the far more advanced treatments introduced in the 1990s [7–12]. As lung function seems to track from childhood to adulthood [9, 13, 14], early onset chronic obstructive pulmonary disease (COPD) is a feared scenario in high-risk subgroups [4, 5].

Better NICU management benefits all infants born preterm, but it also facilitates survival of more immature and vulnerable individuals who may be at particular risk of severe long-term morbidity [2, 15]. Therefore, respiratory health and lung function after EP birth have been closely monitored in population-based controlled longitudinal studies in Western Norway since the 1980s [13, 16]. With the present study, we aimed to address respiratory outcomes at 11 years of age in our most recent cohort born EP in 1999–2000 (EP_1999–2000_) and to compare the findings with those of children born similarly preterm in 1991–1992 (EP_1991–1992_). We hypothesized that changes in perinatal care during the 1990s were associated with respiratory improvements [16, 17].

## Materials and Methods

**Fig 1:**
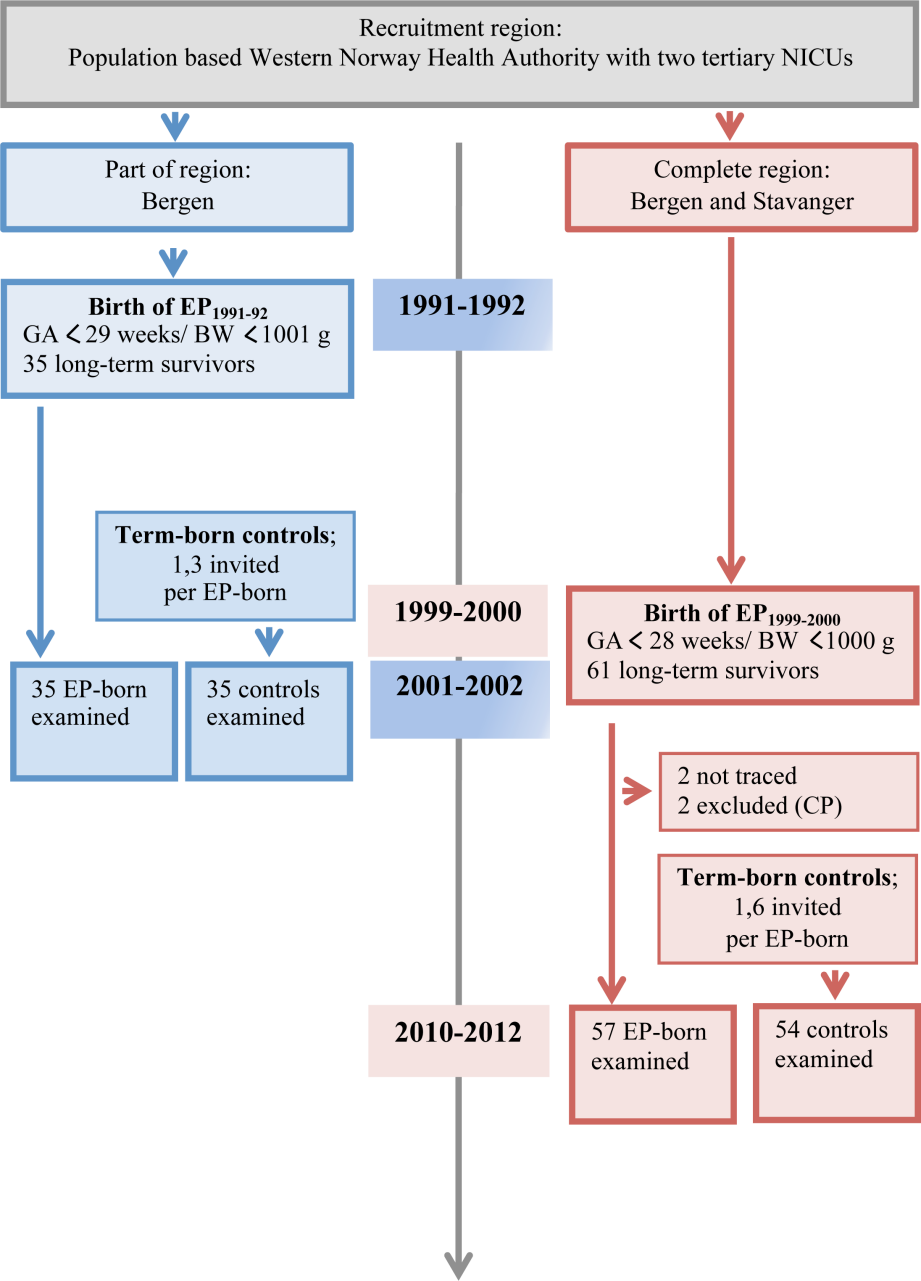
The recruitment of subjects in two cohorts of premature (EP) and matched term-born subjects, one cohort born in 1991–92 and examined in 2001–2002, and one cohort born in 1999–2000 and examined in 2010–2012.

Detailed descriptions are provided in S1 File, and the inclusion process is visualized in Fig 1.

### Subjects, definitions and neonatal background data

The EP_1999–2000_ included all NICU admitted infants born in 1999–2000 with gestational age (GA) <28 weeks or birth weight (BW) <1000 gram within Western Norway Health Authority, which serves a population of approximately 1.1 million. It was a regional selection of a national cohort [2]. The infants were treated at one of the two regional NICUs (Bergen and Stavanger). The EP_1999–2000_ data were compared with data obtained at 10 years of age from all (n = 35) subjects born with GA <29 weeks or BW <1001 gram within a part of the same region (population 615 000) in 1991–92 and treated at the NICU in Bergen (EP_1991–1992_). EP_1991–1992_ data have been published previously in a different context [13, 16] and relevant data are included in the present paper. The acronym ‘EP-born’ is used in this article to represent participants born preterm in both inclusion periods.

For each EP-born participant of both birth-cohorts, the next-born child in the same maternity ward of the same gender with GA >37 weeks and BW >3000 grams were identified from birth protocols and invited as control. If that individual declined, the next-born eligible child was invited until a match was obtained. Background data were extracted from compulsory notifications to the Medical Birth Registry of Norway, registration-forms developed for the study and questionnaires completed by the parents, including the International Study on Asthma and Allergy in Childhood (ISAAC) questionnaire [18]. Small for gestational age (SGA) was defined as BW <10^th^ percentile for GA according to Norwegian growth curves [19]. Z-scores for BW and later anthropometric measures were calculated with reference to Norwegian growth curves [19, 20]. BPD was defined as still dependent on oxygen supplementation at 36 weeks’ postmenstrual age [3]. Oxygen supplementation was given according to similar department policy in the two inclusion periods in the two participating NICUs, and at 36 weeks’ GA it was generally provided through low flow nasal cannulas guided by pulse oximetry targeting 90–95% saturation. Important changes of treatment between the two inclusion periods were more extensive use of surfactant and antenatal corticosteroids and a change from synthetic (Exosurf^®^) (31) to natural surfactant (Curosurf^®^) (Table 1) (32, 33). Most areas of neonatal intensive care medicine had gone through refinements, such as better standardization of antenatal and perinatal care, a higher level of competence among neonatologists and nurses regarding the special needs of these vulnerable infants, and better exploitation of technological advances, such as patient coordinated assisted ventilation and various forms of oscillation.

**Table 1:** Perinatal data comparing children born preterm in 1991–92 (EP_1991–92_) and in 1999–2000 (EP_1999–2000_).

		EP_1991–92_ cohort	EP_1999–2000_ cohort	p-value^a^
**Subjects**; n (%)	Control	35	54	
	All EP	35	57	
	- non BPD	23 (66)	26 (46)	0.067
	- BPD	12 (34)	31 (54)	
**Female gender**;n (%)	Control	22 (63)	25 (46)	0.135
	All EP	22 (63)	28 (49)	0.209
	- non BPD	17 (74)	16 (62)	0.379
	- BPD	5 (42)	12 (39)	0.860
**Birthweight**, gram; mean (SD)	Control	3564 (275)	3701 (434)	0.073
	All EP	933 (204)	850 (175)	0.039
	- non BPD	976 (195)	873 (200)	0.073
	- BPD	851 (203)	831 (151)	0.722
**Birthweight**; sds- score	All EP	-0.36 (0.9)	-0.80 (1.3)	0.054
	- non BPD	-0.37 (0.9)	-0.97 (1.4)	0.076
	- BPD	-0.32 (0.9)	-0.66 (1.2)	0.386
**Gestational age**, weeks; mean (SD)	All EP	26.7 (1.7)	26.8 (1.6)	0.974
	- non BPD	27.2 (1.7)	27.3 (1.6)	0.788
	- BPD	25.8 (1.5)*	26.3 (1.4)**	0.381
**Small for gestational age (SGA)**; n (%)	All EP	5 (14)	20 (35)	0.030
	- non BPD	3 (13)	12 (46)	0.015
	- BPD	2 (17)	8 (26)	0.570
**Postnatal days with oxygen treatment**; median (range)	All EP	49 (2–180)	65 (0–250)	0.109
	- non BPD	34 (2–70)	44 (0–78)	0.254
	- BPD	92 (61–180)	79 (44–250)	0.080
**Ventilator days**; median (range)	All EP	4.0 (0–55)	5.0 (0–24)	0.618
	- non BPD	1.3 (0–40)	2.5 (0–21)	0.212
	- BPD	12.7 (2–55)***	8.0 (0–24)***	0.011
**Antenatal corticosteroids**; n (%)	All EP	15/34 (44)	46 (81)	<0.001
	- non BPD	11 (48)	21 (81)	0.005
	- BPD	4/11 (36)	25 (81)	0.012
**Surfactant**; n (%)	All EP	17 (49)	49/56 (88)	<0.001
	- non BPD	7 (30)	20/25 (80)	0.001
	- BPD	10 (83)	29 (94)	0.367
**Postnatal corticosteroids**; n (%)	All EP	10 (29)	18 (32)	0.772
	- non BPD	2 (9)	1 (4)	0.549
	- BPD	8 (67)	17 (55)***	0.510
**Closing of PDA**; n (%)	All EP	17 (49)	13 (23)	0.013
	- non BPD	7 (30)	3 (12)	0.122
	- BPD	10 (83)	10 (32)	0.004
**Maternal smoking in pregnancy**; n (%)	Control	9 (26)	-	-
	All EP	13 (37)	13/52 (25)	0.205
	- non BPD	10 (43)	5/24 (21)	0.111
	- BPD	3 (25)	8/28 (29)	0.957

Figures are means (SD), medians (ranges) or counts (%).

^**a**^ The p-value denotes differences between those born in 1991–92 and in 1999–2000.

* P-values for group differences between EP non BPD vs. EP BPD within each cohort, * p<0.05,

**p<0.01,

***p<0.001.

The studies were approved by the regional committee on medical research ethics in Western Norway Health Authority (REC West), and parents of all participants gave written consent.

### Measurements

The two birth-cohorts were examined at 10–11 years of age in 2001–2002 and 2010–2012, respectively, using the same type of equipment and the same examination program, except that nitric oxide was not studied in EP_1991–1992_. The children were seen on two separate days at the University Hospitals in Bergen or Stavanger according to place of birth. Spirometry, static lung volumes and pulmonary diffusing capacity for carbon monoxide (*DLCO/KCO*) were measured with Vmax equipment (*SensorMedics Inc*, *Anaheim*, *USA*), applying standard quality criteria [21, 22] with data standardized for age, height and gender [23, 24], except *KCO* reported as raw data. Fractional exhaled nitric oxide (Fe_NO_) was measured with Exhalyzer CLD-88 (*EcoMedics*, *Switzerland*), according to ATS/ERS recommendations [25]. Alveolar NO (ppb) (CA_NO_) and bronchial flux of NO (nl/sec) (Jaw _NO_) were calculated using three different flows (30, 100 and 300 ml/sec) [26]. Methacholine provocation was performed with an inhalation-synchronised dosimetric nebulizer (*Spira Electra*, *Finland*), providing baseline FEV_1_ ≥65% predicted [27, 28]. The test continued until a fall of 20% or more compared to baseline FEV_1_, or until the maximum dose of 11.5 μmol methacholine. Dose-response slope (DRS) was calculated as the ratio of maximum percentage decline in FEV_1_ from baseline to cumulative administered dose of methacholine (%/μmol) [29]. Reversibility to salbutamol was assessed by measuring FEV_1_ before (baseline) and 10–15 minutes after administering 0.1 mg/10 kg salbutamol (*Ventoline*) from a metered dose inhaler via a spacer (*Volumatic*); an increase ≥12% was considered positive response [30]. Methacholine provocation and salbutamol reversibility tests were done on separate days.

### Statistical analysis

Non-paired groups were compared using independent sample t-tests, Mann-Whitney U-test, Fishers exact 2-sided mid-p value [31] or odds ratios (OR), and paired data with the mixed linear model (MLM) of SPSS, as appropriate. For EP_1999–2000_, multiple backward regression models were constructed in order to address potential associations between neonatal data and selected background data (listed in the Results chapter) vs. current FEV_1_. Independent variables were included if the correlation with the dependent variable was > 0.3 and the bivariate correlation with other independent variables was < 0.7. Interaction terms were used to test if effects (differences in lung function between EP-born and matched term-controls) differed between EP-born subgroups; i.e. BPD vs. non BPD, GA-categories (GA ≤25 vs. 26–27 vs. ≥28 weeks) and birth-cohort (1991–1992 vs. 1999–2000). The interaction terms were tested for influence from selected neonatal factors that varied between the two preterm born cohorts (see Results). Providing 60 cases were included, the study had 80% power to detect a difference in z-FEV_1_ of 0.5 between EP_1999–2000_ and matched term-controls, given a two-sided significance level of 0.05. SPSS (version 21.0) was used for computations.

## Results

### Subjects (Fig 1)

In EP_1999–2000_, 61 eligible EP-born were discharged alive, two could not be traced and two were excluded due to cerebral palsy, leaving 57 (93%) participants, all but three Caucasians. In EP_1991–1992_, all 35 eligible EP-born survivors participated, all Caucasians. On average, respectively 1.6 and 1.3 term-born subjects were approached to recruit a full 1:1 control group for the two inclusion periods. In EP_1999–2000_, uneven drop-outs eventually caused a numeric gender difference; i.e., 49% EP-born vs. 46% controls were female (p = 0.85).

### Perinatal data (Table 1)

For subjects admitted to the NICU, survival rates were 81% (EP_1999–2000_) vs. 74% (EP_1991–92_) (p = 0.57). Due to slight differences in the inclusion criteria, mean BW was lower and the proportion born SGA was higher in EP_1999–2000_. Eleven subjects (19%) were included solely by BW (i.e. GA ≥28 weeks) in EP_1999–2000_ (mean GA (range) 29.2 (28–31) weeks), and two solely by BW (i.e. GA ≥29 weeks) in EP_1991–92_ (GA 30 and 31 weeks). The number of participants born at GA ≤27 weeks was 46/57 (81%) and 21/35 (60%), respectively. For subjects with BPD in EP_1999–2000_ compared to EP_1991–1992_, mean GA and BW did not differ, but days on ventilator were fewer, a higher proportion had received prenatal corticosteroids and surfactant treatment and fewer had artificial closure of persistent ductus arteriosus (surgery or indomethacin).

### Anthropometric data and respiratory symptoms (Table 2)

**Table 2:** Anthropometric data and respiratory symptoms at age 11 comparing children born preterm in 1991–92 (EP_1991–92_) and in 1999–2000 (EP_1999–2000_).

		EP_1991–92_ cohort n = 35	EP_1999–2000_ cohort n = 57	p-value^a^
**Age; years**	Control	10.6 (0.4)	11.7 (0.7)	<0.005
	All EP	10.4 (0.4)	11.4 (0.6)	<0.005
	- non BPD	10.4 (0.5)	11.4 (0.6)	<0.005
	- BPD	10.4 (0.4)	11.5 (0.6)	<0.005
**Height**; z-score	Control	0.02 (0.9)	0.00 (1.1)	0.925
	All EP	-0.51 (1.2)*	-0.41 (1.0)*	0.654
	- non BPD	-0.52 (1.3)	-0.38 (1.0)	0.658
	- BPD	-0.49 (1.1)	-0.43 (1.1))	0.868
**Weight**; z-score	Control	0.17 (1.0)	-0.14 (1.1)	0.153
	All EP	-0.48 (1.4)*	-0.33 (1.05)	0.570
	- non BPD	-0.40 (1.7)	-0.43 (1.0)	0.956
	- BPD	-0.61 (0.8)	-0.25 (1.1))	0.303
**BMI**; z-score	Control	0.25 (0.9)	-0.22 (1.0)	0.033
	All EP	-0.28 (1.4)	-0.16 (1.0)	0.644
	- non BPD	-0.18 (1.6)	-0.35 (1.1)	0.660
	- BPD	-0.46 (0.9)	0.00 (1.0)	0.178
**Asthma ever**	Control	3 (9)	5 (9)	0.933
	All EP	12 (34)*	15 (26)*	0.427
	- non BPD	6 (26)	5 (19)	0.587
	- BPD	6 (50)	10 (32)	0.309
**Asthma medication last 12 months**	Control	1 (3)	3 (6)	0.838
	All EP	5 (14)	4 (7)	0.075
	- non BPD	1 (4)	3 (12)	0.775
	- BPD	4 (33)	1 (3)	0.006
**Wheeze last 12 months**	Control	2 (6)	5 (9)	0.587
	All EP	11 (31)*	8 (14)	0.055
	- non BPD	6 (26)	4 (15)	0.383
	- BPD	5 (42)	4 (13)	0.060
**Atopy**	Control	8 (23)	20/50 (40)	0.105
	All EP	9 (26)	12/55 (22)	0.675
	- non BPD	8 (35)	6/25 (24)	0.435
	- BPD	1 (8)	6/30 (20)	0.415

Figures are means (SD), medians (ranges) or counts (%).

^**a**^ The p-value denotes differences between those born on 1991–92 and in 1999–2000.

* P-values for group differences between term controls vs. all EP within each cohort, * p<0.05. Atopy registered as minimum one positive SPT (skin prick test) or IgE test in the EP_1991–92_ cohort and as minimum one positive SPT in the EP_1999–2000_ cohort.

At follow-up, EP_1999–2000_ was one year older than EP_1991–1992_. Anthropometric measures were similar except that the control children for EP_1999–2000_ had slightly lower BMI. The prevalence of ever having been diagnosed with asthma was similar for the two preterm-born cohorts, and the proportion was significantly higher than for their respective term-born groups. Current respiratory symptoms (wheezing) and use of asthma medication tended to be rarer in EP_1999–2000_ than in EP_1991–92_ and not significantly different from term-controls, contrasting EP_1991–92_. Wheeze was unrelated to airflow-limitation, with one report of current wheeze in the overall 15 subjects with z-FEV1 below -1.64, which is the lower limit of normal [23].

### Lung function for EP_1999–2000_

Data on EP_1991–1992_ have been published previously [13, 16], and relevant data are presented in Table C in S1 File. For EP_1999–2000_, data are presented in Tables 3 and 4 and Table D in S1 File. Flow-volume loops were satisfactorily obtained from all participants. Failure rates were minor also for most other lung function tests, except measures of nitric oxide and lung diffusion (12–14 of 57). The failure rates were similar for EP-born and term-controls on the individual tests; details are presented in S1 File. EP-born had lower z-scores for FEV_1_, FEV_1_/FVC and FEF_25–75_, higher airway resistance (Raw), and higher DRS from the methacholine challenge than term-controls. DRS was negatively and similarly associated with z-FEV_1_ (p<0.001) in the EP- and term-born groups (test of interaction, p = 0.21). Static lung volumes (z-TLC, z-FRC, z-RV and RV/TLC), Fe_NO_ and DLCO did not differ significantly between the EP-born and term-born groups. Nine subjects (7 EP-born; 5 BPD and 2 non-BPD, and 2 term-born) had ≥12% increase in FEV_1_ on the salbutamol reversibility test (EP vs. term-born, p = 0.18). Mean FEV_1_ change before vs. after salbutamol did not differ between those born EP and at term, and responses were negatively associated with z-FEV_1_ (p<0.001) in both groups (test of interaction, p = 0.24).

**Table 3:** Lung function variables in 11 year old children born preterm (EP) in 1999–2000 and matched term-born controls, split by neonatal bronchopulmonary dysplasia (BPD).

		Controls N = 54	All EP N = 57	EP non BPD N = 26	EP BPD N = 31	Mean difference (95% CI) All EP vs. Control	* P values EP vs. Control
FEV_1_	*Z*	-0.31(-0.57, -0.04)	-0.65 (-0.90, -0.41)	-0.56 (-0.91, -0.21)	-0.73 (-1.10, -0.37)	-0.35 (-0.70, 0.01)	0.04
FVC	*Z*	-0.16 (-0.42, 0.09)	-0.17 (-0.41, 0.07)	-0.17 (-0.48, 0.13)	-0.17 (-0.54, 0.20)	-0.01 (-0.36, 0.33)	0.96
FEV_1_/FVC	*Z*	-0.30 (-0.54, -0.05)	-0.80 (-1.07, -0.54)	-0.69 (-1.06, -0.31)	-0.90 (-1.29, -0.52)	-0.52 (-0.89, -0.16)	0.005
FEF_25–75_	*Z*	-0.53 (-0.79, -0.27)	-1.14 (-1.39, -0.89)	-1.04 (-1.40, -0.68)	-1.22 (-1.58, -0.87)	-0.63 (-0.98, -0.27)	0.001
Raw	*Z*	0.68 (0.51, 0.85)	1.23 (0.77, 1.68)	0.96 (0.72, 1.21)	1.45 (0.61, 2.29)	0.54 (0.05, 1.04)	0.58
Reversibility^a^	*% change (FEV*_*1*_ *)*	5.0 (3.9, 6.1)	6.6 (4.3, 8.8)	5.2 (2.9, 7.4)	7.8 (4.0, 11.5)	1.6 (-0.9, 4.1)	0.18
DRS^b^	*Geometric mean*	3.47 (2.19, 5.50)	13.18 (8.16, 21.37)	11.48 (5.75, 23.44)	14.79 (7.24, 29.51)	3.80 (2.00, 7.24)	<0.001
TLC	*Z*	0.45 (0.13, 0.76)	0.30 (0.05, 0.55)	0.17 (-0.11, 0.46)	0.41 (0.00, 0.82)	-0.15 (-0.55, 0.25)	0.32
FRC	*Z*	-0.34 (-0.74, 0.06)	0.07 (-0.27, 0.41)	-0.26 (-0.67, 0.15)	0.36 (-0.16, 0.89)	0.41 (-0.11, 0.92)	0.06
RV	*Z*	0.27 (-0.09, 0.63)	0.003 (-0.30, 0.30)	0.05 (-0.40, 0.51)	-0.04 (-0.47, 0.38)	-0.27 (-0.73, 0.20)	0.17
RV/TLC	*Ratio*	26.2 (24.4, 27.9)	25.9 (24.0, 27.9)	26.9 (24.0, 29.8)	25.2 (22.4, 27.9)	-0.2 (-2.8, 2.4)	0.75
DLCO	*% predicted*	88.2 (84.3, 91.7)	86.5 (80.6, 92.4)	87.4 (81.4, 93.3)	85.7 (75.0, 96.4)	-1.6 (-8.5, 5.2)	0.68
VA	*% predicted*	97.1 (91.4, 102.9)	97.8 (91.8, 103.7)	92.6 (83.7, 101.4)	102.7 (94.7, 110.8)	0.6 (-7.5, 8.7)	0.65
KCO	*Mmol/kPa*.*min*	1.74 (1.66, 1.82)	1.69 (1.61, 1.77)	1.77 (1.67, 1.87)	1.61 (1.48, 1.74)	-0.05 (-1.6, 0.06)	0.32
	*% predicted*	80.0 (76.7, 83.3)	76.3 (72.5, 80.2)	80.0 (75.3, 84.8)	72.8 (66.9, 78.8)	-3.6 (-8.6, 1.3)	0.13
Fe_NO 0.05_	*G*. *mean*	11.77 (9.53, 14.54)	9.65 (7.97, 11.69)	10.46 (7.59, 14.43)	9.08 (7.08, 11.65)	-1.22 (-1.62, 1.09)	0.18
C_A_NO	*ppb*	1.48 (1.22, 1.74)	1.25 (0.97, 1.54)	1.30 (0.85, 1.74)	1.22 (0.82, 1.61)	-0.16 (-0.56, 0.25)	0.24
J_aw_NO	*nl/sec*	0.77 (0.57, 0.96)	0.86 (0.54, 1.17)	0.83 (0.47, 1.19)	0.88 (0.36, 1.41)	0.09 (-0.28, 0.46)	0.62
Fe_NO nasal_	*ppb*	805 (696, 915)	825 (745, 905)	901 (758, 1044)	764 (675, 854)	20 (-115, 155)	0.73

Figures are observed means (95% confidence intervals), unless otherwise stated. For abbreviations, please see list. Lung diffusion data reported as % predicted and as raw-data, and nitric oxide (NO) data only as raw-data, due to suboptimal reference equations for children.

* Interaction terms testing differences between EP and matched term-born controls in the group with BPD vs. without BPD were non-significant for all variables, and therefore only the p-values for all EP vs. term-born controls were reported.

^a^ Reversibility is given as percentage change in FEV_1_ after vs. before administration of beta agonist, assuming the pre-value is baseline.

^b^ DRS is the ratio of maximum percentage decline in FEV_1_ from baseline to cumulative administered dose (μmol) of methacholine (%/μmol), reported as geometric means due to a highly skewed distribution.

**Table 4:** Lung function variables reflecting airway flow before and after administration of salbutamol, and bronchial responsiveness to methacholine, in 11 year old children born preterm (EP) in 1999–2000, split by gestational age (GA) at birth.

		Controls N = 54	GA ≤ 25 weeks N = 10	GA 26–27 weeks N = 36	GA ≥ 28 weeks N = 11
Gestational age (SD)	*weeks*	-	24.4 (0.5)	26.7 (0.5)	29.2 (1.0)
Birth weight (SD)	*grams*	-	741.2 (107)	904.1 (177)	770.1 (146)
BW z-scores (SD)		-	0.09 (0.9)	-0.54 (1.0)	-2.46 (0.7) ***
No. with SGA^a^	*(% of group)*		1 (10)	8 (22)	11 (100)
No. with BPD	*(% of group)*	-	8 (80)	20 (56)	3 (27) ***
FEV_1_	*z*	-0.27 (-0.52, -0.01)	-0.46 (-1.28, 0.37)	-0.54 (-0.82, -0.27)	-1.22 ** (-1.84, -0.59)
	*z-Post-β*_*2*_	-0.07 (-0.34, 0.21)	0.05 (-1.04, 1.13)	-0.41 (-0.82, 0.01)	-0.67 (-1.29, -0.04)
FEV_1_/FVC	*z*	-0.30 (-0.54, -0.05)	-0.13 (-0.98, 0.73)	-0.82 * (-1.12, -0.52)	-1.38 ** (-1.93, -0.83)
	*z- Post-β*_*2*_	0.22 (0.00, 0.44)	0.39 (-0.39, 1.18)	-0.23 (-0.58, 0.13)	-0.31 (-0.89, 0.28)
FEF_25–75_	*z*	-0.52 (-0.78, -0.27)	-0.92 (-1.59, -0.25)	-1.07 * (-1.37, -0.77)	-1.78 ** (-2.34, -1.22)
	*z- Post-β*_*2*_	0.06 (-0.20, 0.31)	-0.07 (-0.88, 0.74)	-0.58 (-0.91, 0.24)	-0.50 (-1.17, 0.17)
DRS	*geometric mean*	3.48 (2.21, 5.47)	3.58 (1.11, 14.19)	12.74 ** (6.95, 23.33)	38.54 ** (1.74, 85.31)

Figures are observed means (95% confidence intervals) split by GA categories for those lung function variables that differed between the EP and term-born groups. For abbreviations, please see list. Interaction terms, testing if differences between EP and matched term-born controls were different over the three GA categories, were non-significant for all variables. * p-values testing differences between EP-born subgroups, or (when possible) EP-born subgroups vs. control subjects,

* p<0.05,

** p<0.01,

*** p<0.001.

^a^ SGA = small for gestational age.

There was no effect of neonatal BPD vs. no BPD on any of the assessed lung function variables (non-significant interaction-terms). Lung function variables that differed between the EP- and term-born groups were analyzed also by GA-category, and the deficits were numerically most pronounced for the GA-category ≥28 weeks, although interaction terms were non-significant (Table 4).

### Lung function related to perinatal and background variables

For EP_1999–2000_, BW z-score was the only significant predictor of z-FEV_1_ at 11 years (B = 0.24; p = 0.04; R^2^ = 0.10) in a regression model including the following perinatal variables: GA, BW z-scores, days on ventilator, days on oxygen treatment, use of antenatal and postnatal corticosteroids, surfactant treatment and BPD vs. no-BPD. Applying the same model on the EP_1991–1992_, BW z-scores similarly predicted z-FEV_1_ (B = 0.35; p = 0.02) in a final model; however, including also days of oxygen (B = -0.011; p<0.001) and antenatal corticosteroids (B = 0.59; p = 0.03) (R^2^ = 0.48, all three variables). Maternal smoking in pregnancy was not associated with z-FEV_1_ at age 11. Regressing the background variables asthma ever, asthma medication last 12 months, wheeze last 12 months, atopy and maternal smoking in pregnancy on z-FEV_1_ at age 11, revealed no significant associations for either EP or term-born in any birth-cohort.

### Differences between EP_1991–1992_ and EP_1999–2000_

**Fig 2:**
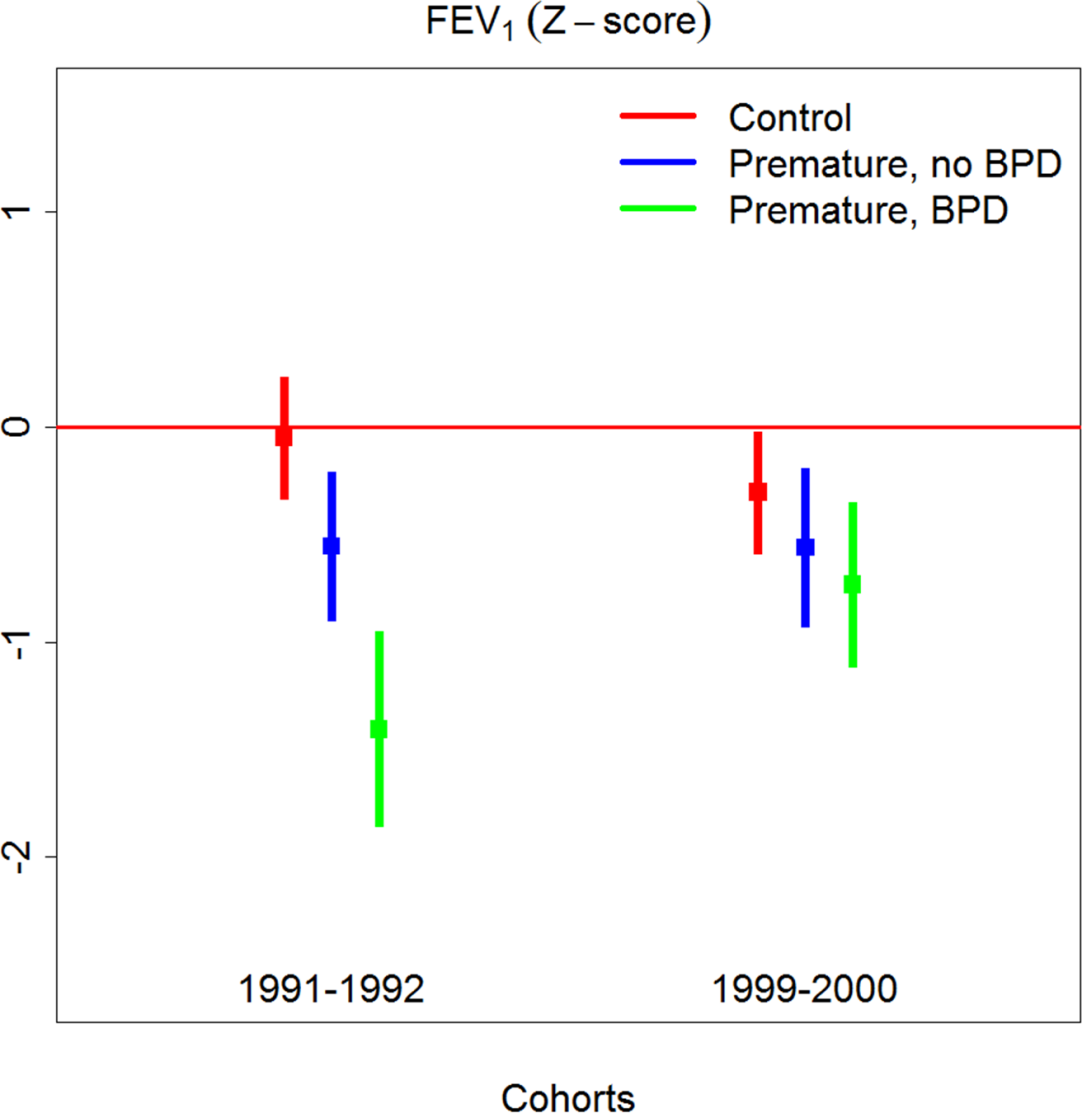
Z-FEV_1_ in 11 year old term and preterm-born (EP) subjects born in 1991–92 and 1999–2000, EP-born split by the presence of BPD.

**Fig 3:**
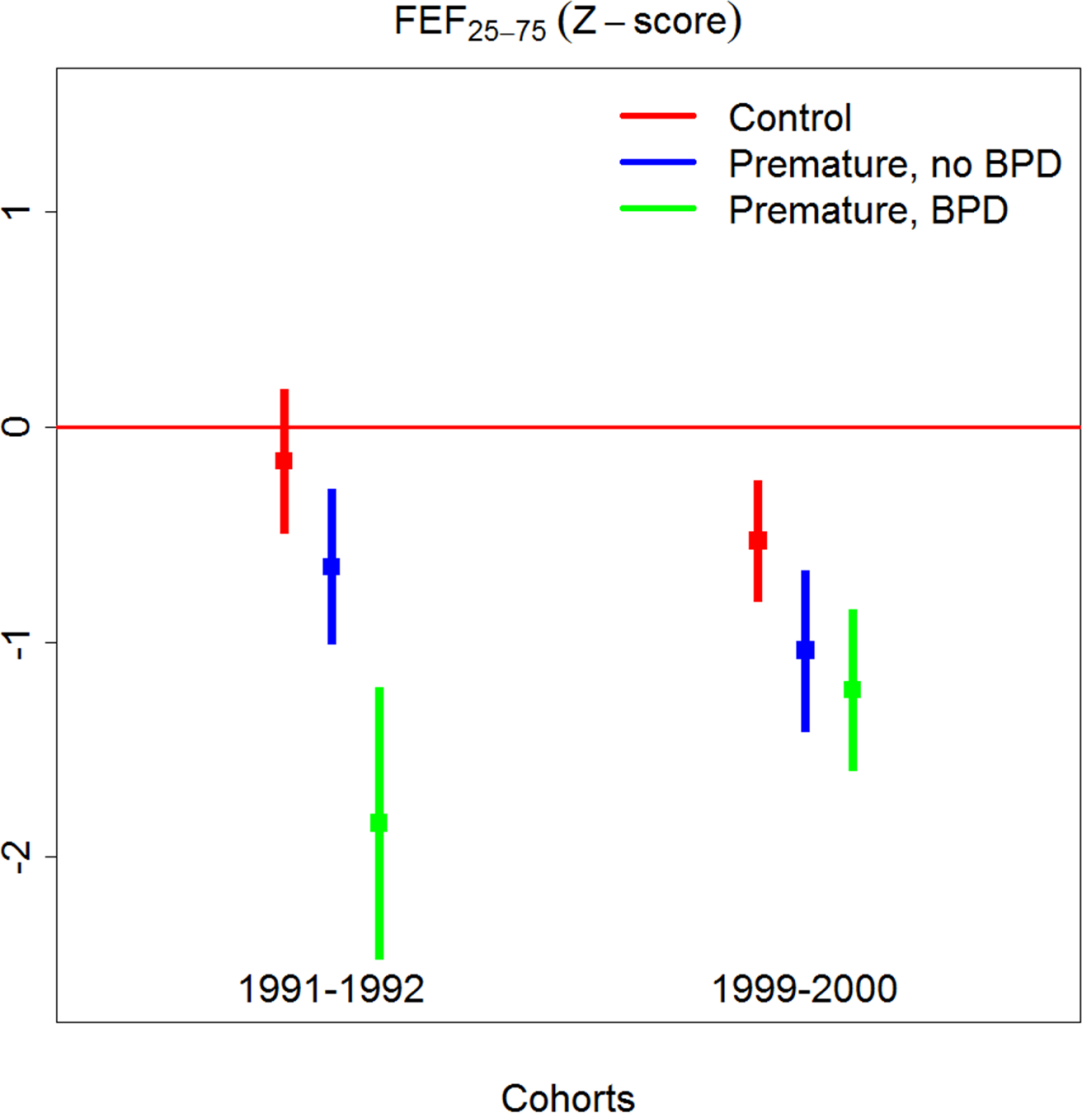
Z-FEF_25–75_ in 11 year old term and preterm-born (EP) subjects born in 1991–92 and 1999–2000, EP-born split by the presence of BPD.

Paired differences between EP-born and matched term-controls for the inclusion periods 1991–1992 and 1999–2000 are given in Table 5 and illustrated in Figs 2 and 3. The differences were significantly smaller for the variables z-FEV_1_, z-FVC and z-FEF_25–75_ and RV/TLC for the group *with BPD* born in 1999–2000 compared to the group *with BPD* born in 1991–1992 (tests of interaction). Adjusting these interaction terms for differences between the study periods regarding GA, BW, days on ventilator and PDA management did not influence conclusions, whereas the effect disappeared for z-FEV_1_ when surfactant and/or antenatal corticosteroids were included in the model. Differences between EP-born *without BPD* and their matched term-controls did not differ between two inclusion periods.

**Table 5:** Comparison of lung function indices in two cohorts of children born preterm in 1991–92 (EP_1991–92_) and in 1999–2000 (EP_1999–2000_).

	EP_1991–1992_ cohort			EP_1999–2000_ cohort			
	Control vs. all-EP	Control vs. EP no-BPD	Control vs. EP BPD	Control vs. all-EP	Control vs. EP no-BPD	Control vs. EP BPD	* p-value, control vs. EP BPD
FEV_1_; z-score	0.86 (0.44, 1.26)	0.53 (-0.00, 1.07)	1.46 (0.80, 2.13)	0.35 (-0.01, 0.70)	0.25 (-0.30, 0.79)	0.43 (-0.08, 0.94)	0.02
FVC; z-score	0.44 (0.07, 0.82)	0.41 (-0.11, 0.93)	0.51 (-0.14, 1.16)	0.01 (-0.33, 0.36)	0.01 (-0.53, 0.54)	0.01 (-0.49, 0.52)	< 0.001
FEV_1_/FVC; z-score	0.57 (-0.03, 1.18)	0.08 (-0.60, 0.77)	1.51 (0.65, 2.36)	0.52 (-0.16, 0.89)	0.41 (-0.15, 0.97)	0.62 (0.09, 1.15)	0.03
FEF_25–75_; z-score	0.94 (0.47, 1.42)	0.54 (-0.07, 1.14)	1.72 (0.96, 2.47)	0.63 (0.27, 0.98)	0.52 (-0.02, 1.06)	0.72 (0.20, 1.23)	0.04
TLC; z-score	0.10 (-0.26, 0.45)	0.24 (-0.24, 0.72)	-0.18 (-0.76, 0.43)	0.15 (-0.25, 0.55)	0.28 (-0.33, 0.88)	0.04 (-0.54, 0.62)	0.74
RV; z-score	-0.17 (-0.56, 0.21)	0.16 (-0.33, 0.65)	-0.79 (-1.39, -0.19)	0.27 (-0.20, 0.73)	0.21 (-0.49, 0.92)	0.31 (-0.37, 0.99)	0.02
RV/TLC	-1.27 (-4.94, 0.11)	-0.59 (-3.93, 2.74)	-5.77 (-9.87, -1.66)	0.21 (-2.40, 2.82)	-0.69 (-4.67, 3.28)	1.01 (-2.82, 4.84)	0.03
DRS; geometric mean	5.01 (2.19, 11.22)	3.47 (1.17, 10.47)	11.22 (2.69, 47.86)	3.80 (2.00, 7.24)	3.31 (1.20, 8.71)	4.27 (1.62, 10.96)	0.36

Figures are mean differences (95% confidence intervals) between EP born subgroups and their respective matched term-born control subjects. The units are z-scores, except the ratio RV/TLC and the variable DRS where figures are differences in the dose response slope to methacholine. Positive figures indicate higher z-scores for the control group or higher DRS for the EP-born group.

* Interaction terms, testing if differences between EP and matched term-born controls were different in the two study periods (1991–92 vs. 1999–2000) were non-significant, except for the groups with neonatal BPD, for which p-values are given.

## Discussion

This study showed that respiratory outcomes in mid-childhood were encouraging in children born at extremely low GAs or BWs in 1999–2000. Still, the preterm born participants had more small-airway obstruction and bronchial hyperresponsiveness than matched term-controls, but static lung volumes, diffusing capacity and exhaled nitric oxide did not differ. Outcome data were in most respects unrelated to BPD, and those born most immature did surprisingly well. Compared to children born similarly preterm in the same region in 1991–1992, lung function data were generally better, and particularly for those with neonatal BPD, where significant improvements had occurred for important variables.

### Strengths and limitations

The major strengths of this study were the population-based design with almost complete attendance, and recruitment of matched control groups that followed a strict algorithm based on the ‘next-born-subject’ principle, minimizing risks of selection bias. An age difference of approximately one year at follow-up between the two birth-cohorts was adjusted for by reference equations or by statistical models. Potential bias introduced by a two-center design was limited by statistical analyses performed according to the matched preterm versus term-born structure. The algorithm for inclusion was based on a combined use of GA and BW, initially preferred in 1991–1992 to ensure inclusion of all children perceived to be extremely immature at birth. We continued this approach in 1999–2000, although changing the GA criterion from <29 weeks to <28 weeks. Due to the inclusion criteria the data may not be generalizable to subjects born extremely preterm in general. A diagnosis of BPD must reflect oxygen treatment algorithms, which to some extent is subject to department policy. In this study, the same senior neonatologists operated the same algorithms for oxygen supplementation in both inclusion periods, making systematic changes unlikely. The study could not discriminate moderate from severe BPD since oxygen treatment at 36 weeks GA was provided through low flow nasal cannulas without recording the exact fraction of inspired oxygen (FiO_2_) [3]. By nature, unfavourable events or conditions tend to be linked in neonatal intensive care medicine, often setting up vicious circles that require treatments with long-term side effects. Observational statistical models struggle with these scenarios due to collinearity between variables. To limit spurious associations we therefore excluded the independent neonatal variable considered least meaningful if a correlation coefficient was ≥ 0.7 in the same analysis. This situation calls for cautious interpretation of regression models that aim to address statistical associations between such variables and outcome. The study may be criticized for having few participants. Longitudinal studies over decades are by nature difficult to perform as demonstrated by the paucity of similar data. The power calculations indicate reasonable detection limits.

### Lung function in the children born preterm in 1999–2000

The lung function abnormalities in the 11 year old children born preterm in 1999–2000 were minor. FEV_1_ was mostly within normal limits and they had no signs of abnormal volume distribution or diffusing capacity for carbon monoxide. However, forced mid-expiratory flow (FEF_25–75_) in the range of 75% predicted and significant methacholine hyperresponsiveness indicate that small airway airflow limitation and bronchial abnormalities were still present. Those born most immature did surprisingly well, in that lung function variables did not differ significantly from those of the term-controls. Moreover, the presence of neonatal BPD was not significantly associated with outcome, contrasting some [12, 32], and consistent with one previous study [11]. Most of the differences between preterm and term-born groups seemed to be explained by the *highest* GA-category; i.e. basically SGA infants with GA ≥28 weeks who were included on the basis of BW <1000 gram, in whom significant airway obstruction and remarkably high bronchial hyperresponsiveness were observed. In adjusted regression models, BW z-score was the only remaining neonatal variable associated with FEV_1_ at age 11. Thus, within the frames of todays advanced NICU management, the paradigm that neonatal BPD or extreme immaturity is inevitably linked to poor long-term pulmonary outcome may need to be revised. However, statements regarding causal pathways for novel findings are bound to be speculations within the frames of a study of this kind.

Interestingly, administration of salbutamol increased all airflow variables to within 0.5 z-score of zero; i.e. clearly within normal limits. Salbutamol responses were negatively and similarly associated with FEV_1_ in the EP-born and term-control group, further reducing the group differences. Few subjects had salbutamol responses considered clinically significant (≥12% from baseline) [30], and on average the responses did not differ between the EP- and term-born group. The question to what extent airway obstruction after EP birth is a fixed or reversible phenomenon therefore remains unanswered.

Pulmonary abnormalities after EP birth may clinically mimic pediatric asthma, and studies have reported increased respiratory symptoms and more hospital admissions, particularly during the first few years of life [33–35]. In this group of 11 year old children born EP in 1999–2000, current respiratory symptoms and use of asthma medication were reassuringly rare, also in those who were most immature at birth, contrasting some previous studies (8, 12, 36). Childhood asthma is generally characterized by eosinophilic airway inflammation (17), which may be assessed by fractional exhaled nitric oxide (FeNO) (25), with extended FeNO analyses as a new promising tool (26). We found that neither FeNO nor alveolar NO differed between the preterm and term-born group, providing support for the notion that eosinophilic airway inflammation is not involved in lung disease after EP birth (10, 37). However, active inflammatory mechanisms may still be involved, and recent studies have indicated increased oxidative stress in the respiratory system after EP birth, pathways this study was not set up to explore (38, 39).

Some authors have expressed optimism regarding long-term pulmonary outcomes in children who were born EP more recently [11, 36]. Kotecha et al. found in their review milder impairments in FEV_1_ for preterm born children with neonatal BPD (defined by need of oxygen treatment at 28 days of age) in recent compared to earlier studies [36]. However, two research groups reported data indicating that respiratory abnormalities are still present after EP birth; one regional study of children born at GA < 28 weeks or BW < 1000 grams in 1997 in the state of Victoria, Australia [12, 32], and another based on the EPICure study of children born atGA < 26 weeks in 1995 in the UK or Ireland [10, 32]. Both studies reported significantly lower z-FEV_1_ and z-FEF_25–75_ in preterm compared to term-born children. The Australian study [12] compared children born in 1997 with a group born in 1991–92, and found overall airflow limitation in both groups, but for those with no history of neonatal BPD, the children born in 1997 had better z-FEV_1_ and z-FVC than those born in 1991–92. In light of the possibility that more children at risk of unfavorable outcome may have survived in the most recent cohort, this may be interpreted as a positive development. These findings are in line with ours, although we found this positive development particularly evident in the group *with* neonatal BPD. The EPICure study had more extreme inclusion criteria, with mean GA at birth almost two weeks lower (24.9) and BW approximately 100 gram lower (750 gram) than the EP_1999–2000_ cohort of our study, and also a higher rate of neonatal BPD; i.e. 70% compared to 50%. They found no differences between preterm and term-born participants regarding FRC, TLC and alveolar volume, which is comparable to our findings, but elevated RV/TLC and some impairments in DLCO and DLCO/VA; differences that we did not see as clearly in our EP_1999–2000_ cohort but resembling our findings at ten years of age in the EP_1991–1992_ cohort [16]. One may speculate that more infants born at the limits of viability in the EPICure study may have contributed to these differences in findings.

### Comparing lung function of children born preterm in 1991–1992 and 1999–2000

There were significant improvements for z-FEV_1_ and other important lung function variables from 1991–1992 to 1999–2000. This was particularly evident for the group of children with neonatal BPD, who presumably had the most turbulent neonatal history. The interaction terms used to assess these improvements were robust for adjustment for most perinatal variables, except that use of antenatal corticosteroids and surfactant eliminated the improvements in z-FEV_1_. Thus, statistical modeling of the dataset suggested that more extensive use of these modalities may partly explain the observed improvement with time. However, a limitation that applies to studies of this kind is that a variety of known and unknown influences of possible significance for outcome cannot be accounted for in the applied regression models. Thus, more and larger studies are required to resolve this issue.

The inclusion algorithms varied slightly between the two preterm-born cohorts. Despite this, the two groups had fairly similar BWs and GAs, although with more SGA children included in 1999–2000. More SGA children and more extreme criteria as regards GA in 1999–2000 should theoretically lead to worse outcomes, whereas the opposite was in fact observed. Thus, one may argue that this *strengthens* the notion that outcome did in fact improve for the average infant born preterm during the 1990s.

The field of neonatal intensive care is in constant change, and it has been suggested that pushing the limits of viability might have masked improvements in outcome [4], although some recent studies have indicated otherwise [12, 37]. We have previously reported respiratory health and lung function data for subjects born EP in the early 1980s and 1990s and suggested improvements with time in groups *without* neonatal BPD [13]. The present study which also included children born in this millennium, indicates that improvements has also occurred in children *with* BPD, i.e. the infants with the most turbulent neonatal history.

## Conclusion

Eleven year old children born at extremely low GAs or BWs in 1999–2000 had more small-airway obstruction and bronchial hyperresponsiveness than matched term-controls, but lung volumes and diffusing capacity were similar. Outcome was mostly unrelated to BPD, and those born most immature did surprisingly well. Compared to a group born similarly preterm in the same region nearly one decade earlier, lung function was generally better, particularly after neonatal BPD. The findings indicate that infants born preterm in this millennium may have better pulmonary prognosis than previously assumed, and that BPD or extreme immaturity may not necessarily be linked to poor lung function in mid-childhood.

## Supporting Information

S1 File (DOC)

## Abbreviations

BW: birthweight
CA_NO_: alveolar NO
COPD: chronic obstructive pulmonary disease
CPAP: continuous positive airway pressure
DLCO: lung diffusion capacity for carbon monoxide
DRS: dose-response-slope
EP: preterm born participants of the present study
ERS: European Respiratory Society
FE_NO_: fractional exhaled nitric oxide
FEV_1_: forced expiratory volume in 1 second
FEF_25–75_: forced expiratory flow between 25 and 75% of vital capacity
FRC: functional residual capacity
FVC: forced vital capacity
GA: gestational age
Hb: haemoglobin
Jaw_NO_: bronchial flux of NO
KCO: diffusion capacity corrected for alveolar volume
NEC: necrotizing enterocolitis
NICU: neonatal intensive care unit
PDA: persistent ductus arteriosus
PD20: the dose methacholine required to lower FEV1 ≥ 20% of baseline level
R_aw_: airway resistance
ROP: retinopathy of prematurity
RV: residual volume
SGA: small for gestational age
SPT: skin prick test
TLC: total lung capacity
VA: alveolar volume
