# Supplementary material for: Children Born Preterm at the Turn of the Millennium Had Better Lung Function Than Children Born Similarly Preterm in the Early 1990s
Source: PLoS One. 2015 Dec 7;10(12):e0144243. doi: 10.1371/journal.pone.0144243 (PMC4671691; doi:10.1371/journal.pone.0144243)
Supplement: S1 File — (DOC) [file pone.0144243.s001.doc]

**Children born preterm at the turn of the millennium had better lung function than children born similarly preterm in the early 1990s**

**S1 Supporting information**

# Materials and Methods

**Background information**

During the period from the early to the late 1990s, there was an increasing use of surfactant and a change from synthetic (Exosurf) to the presumably better natural surfactant (Curosurf), and also more extensive use of antenatal corticosteroids This was noted in the main text of this article. Furthermore, the 1990s were characterized by a large number of relatively small changes in NICU care, more than by single big leaps in development. Most areas of neonatal intensive care medicine had gone through refinements, such as better standardization of antenatal and perinatal care, a higher level of competence among neonatologists and nurses regarding the special needs of these vulnerable infants, and better exploitation of technological advances, such as patient coordinated assisted ventilation and various forms of oscillation. In 1991-1992 oscillation had recently been introduced with ventilators capable of exploiting this technique, in our department Infant Star and SensorMedics. Seven years later these techniques were still in use, although with even more advanced ventilators, and better techniques for patient coordinated ventilation had been developed. Moreover, the skills with which these techniques were used had improved, based on the improved knowledge that had accumulated during the 1990s.

**Subjects, data collection and definitions**

Asthma medication included inhaled corticosteroids and short or long acting beta 2 agonists (in separate or combined device), anticholinergics and oral leukotriene modifiers (Singulair®). Atopy was defined as minimum one positive skin prick test (SPT) in a panel of relevant airway allergens. Maternal smoking in pregnancy was defined by self-reported daily or occasional smoking during pregnancy with the index subject.

Current height (cm) was measured with a stadiometer and weight (kg) with an electronic weight, and standardized for gender and age [1]. The age was calculated as the difference between the examination date and the birth date using the automated method provided by SPSS, with values entered into the reference equations using two decimals.

**Lung function measurements**

Subjects born in 1999-2000 (EP_1999-2000_) were seen twice in 2010-2012 at the University Hospitals in Bergen or Stavanger, according to place of birth, with pediatric examination, comprehensive lung function tests, skin prick tests for allergy and anthropometric measurements. Subjects born in 1991-1992 (EP_1991-1992_) went through similar examinations (except nitric oxide measurements) using similar equipment and testing procedures in 2001-2002. Subjects were rescheduled if respiratory symptoms suggestive of an obstructive exacerbation or a viral infection were suspected during the past two weeks. Inhaled corticosteroids and short-acting beta 2 agonists were stopped 1 day prior to testing, and long-acting beta 2 agonists and leukotriene modifiers 2 days prior to testing.

Spirometry and pulmonary diffusing capacity for carbon monoxide (*D*LCO) (single breath method) were measured with Vmax 22 equipment (*SensorMedics Inc, Anaheim, USA*), static lung volumes with V6200 Autobox Body Plethysmograph (*SensorMedics Inc, Anaheim, USA*), all in sitting position, wearing a nose clip, applying standard quality criteria [2-6]. The spirometry values from the second test day were used, for those subjects (2 EP and 3 term-born) that declined (or were unable to perform) after the first day, values from the first test day were used. Measurements were standardized for age, height and gender [7, 8], and KCO reported as raw-data. Variables recorded were forced expiratory volume in one second (FEV_1_), forced vital capacity (FVC), forced expiratory flow between 25% and 75% of vital capacity (FEF_25-75_), total lung capacity (TLC), functional residual capacity (FRC), residual volume (RV), airway resistance (Raw), diffusion capacity for CO (DLCO), alveolar volume (VA), and diffusion capacity adjusted for alveolar volume (KCO).

Fractional exhaled nitric oxide (Fe_NO_) at an exhalation rate of 50 ml/sec was measured with Exhalyzer CLD-88 (*EcoMedics, Switzerland*), according to ATS/ERS recommendations [9]. The test was commenced before spirometry and the other lung function tests. NO-free air was inhaled to near total lung capacity, followed immediately by full exhalation through a dynamic flow restrictor with a target flow of 50 ml/second for at least 6 seconds, in standing position, without wearing a nose clip. Fe_NO_ (ppb) was calculated as the mean value from 3 measurements with coefficient of variation (CV) within 10% acceptability, or if this was not applicable, from the curve with the most stable and horizontal plateau phase of expiration. If the subjects had measures from both test days, mean values from the two days were used for statistical analysis. Fe_NO_ values were in statistical analyses considered log-normally distributed and reported as geometric means. Mathematical models can be used to differentiate the sites of NO production in the respiratory system, specifically bronchial vs. alveolar NO [10, 11]. Alveolar NO (ppb) (CA_NO_) and bronchial flux of NO (nl/sec) (Jaw _NO_) were calculated using three different flows (30, 100 and 300 ml/sec), and nasal Fe_NO_ was measured, by exhalation through a nasal prong.

Methacholine provocation (PD20) was performed with an inhalation-synchronised dosimetric nebulizer (*SPIRA Electra, Finland*), providing baseline FEV_1_ ≥65% of predicted [12, 13]. Baseline lung function measurements were obtained in sitting position after saline inhalation, followed by inhalation of doubling doses of methacholine via the *SPIRA* dosimeter with controlled tidal breathing according to ATS guidelines. The children inhaled at a flow 0.5 l/s, the aerosolisation started when 100 ml air was inhaled from functional respiratory capacity and the aerosol delivery time was set to 0.5 seconds. The first dose was 0.05 μmol methacholine and the test continued until a fall ≥20 % compared to post-saline (baseline) FEV_1_, or until the maximum dose of 11.5 μmol methacholine was reached. Dose-response slope (DRS) was calculated as the ratio of maximum percentage decline in FEV_1_ from baseline to cumulative administered dose (µmol) of methacholine (%/µmol); in statistical analyses considered log-normally distributed and reported as geometric means [14]. Reversibility to salbutamol was given as percentage change in FEV_1_ after vs. before administration of 0.1 mg/10 kg salbutamol (*Ventoline*) from a metered dose inhaler via a spacer (*Volumatic*), assuming the pre-value as baseline. The test was commenced on a separate day from the PD20 test.

Reversibility to salbutamol was assessed by measuring FEV_1_ before (baseline) and 10-15 minutes after administering 0.1 mg/10 kg salbutamol (*Ventoline*) from a metered dose inhaler via a spacer (*Volumatic*), an increase ≥12% ((FEV_1_ value after - FEV_1_ value before) x 100/ FEV_1_ value before) was considered positive response [15].

Skin prick tests for house dust mite (*D. Farinae* and *D. Pteronyssinus*), animal dander (cat, dog, horse), pollens (timothy, birch and mugwort) and moulds (*Alternaria* and *Cladesporium*) were done with standard extracts (Soluprick®SQ, ALK-Abello AS, Hørsholm, Denmark) in accordance with European guidelines[16]. Histamine (10 mg/mL) and the allergen diluent were used as positive and negative controls. A reaction was judged positive if mean of the two perpendicular weal diameters was at least 3.0 mm.

**Statistical methods**

Means with standard deviations (SD) or 95% confidence intervals (95% CI), medians with ranges, counts with group percentages were calculated. Groups were compared by independent sample t-test, Mann-Whitney U-test, Fisher’s exact 2-sided mid-p value or odds ratios (OR), as appropriate. Paired data were compared with the mixed linear model of SPSS, allowing for contributions also from pairs with missing data, using interaction terms to test if effects or differences differed between the various subgroups of the study, that is neonatal BPD vs. no BPD, GA categories (GA ≤25 vs. 26-27 vs. ≥28 weeks) and birth-cohorts (EP_1999-2000_ vs. EP_1991-1992_), a positive interaction would mean that the difference between term-born and EP-born would differ between either BPD groups, GA groups or birth cohorts. The study had 80 % power to detect a difference in FEV_1_ z-scores of 0.50 if 60 cases were included in the EP_1999-2000_ cohort, providing a two-sided significance level of 0.05.

Multiple backward regression models were constructed to address potential associations between perinatal data or background data vs. lung function data at age 11, primarily with z-FEV_1_ as outcome as it is considered the most robust index of airway obstruction. Variables were entered into the regressions if their individual association with the dependent had a p-value ˂0.1.

SPSS (version 21.0) was used for computations.

# Results

**Subjects**

Two of the 61 children born EP in 1999-2000 could not be traced, two were excluded due to severe cerebral palsy, none of whom were able to cooperate when doing lung function testing, and a third child was unable to cooperate to other than spirometry. All but three participating subjects were Caucasian.

**Table A**: Perinatal data comparing children born preterm in 1991-92 (EP_1991-92_) and in 1999-2000 (EP_1999-2000_).

|  |  | **EP_1991-92_ cohort** | **EP_1999-2000_ cohort** | **Cohort difference** | **p-value^a^** |
| --- | --- | --- | --- | --- | --- |
| **Subjects;** n (%) | Control | 35 | 54 |  |  |
|  | All EP | 35 | 57 |  |  |
|  | - non BPD | 23 (66) | 26 (46) | 20 (-4, 44) | 0.067 |
|  | - BPD | 12 (34) | 31 (54) | 20 (-13, 53) |  |
| **Female gender;** n (%) | Control | 22 (63) | 25 (46) | 17 (-4, 38) | 0.135 |
|  | All EP | 22 (63) | 28 (49) | 14 (-7, 35) | 0.209 |
|  | - non BPD | 17 (74) | 16 (62) | 12 (-14, 38) | 0.379 |
|  | - BPD | 5 (42) | 12 (39) | 3 (-30, 36) | 0.860 |
| **Birthweight,** gram; mean (SD) | Control | 3564 (275) | 3701 (434) | 137 (-28, 301) | 0.073 |
|  | All EP | 933 (204) | 850 (175) | -84 (-163, -4) | 0.039 |
|  | - non BPD | 976 (195) | 873 (200) | -104 (-218, 10) | 0.073 |
|  | - BPD | 851 (203) | 831 (151) | -20 (-134, 94) | 0.722 |
| **Birthweight;** sds- score | All EP | -0.36 (0.9) | -0.80 (1.3) | -0.44 (-0.93, 0.04) | 0.054 |
|  | - non BPD | -0.37 (0.9) | -0.97 (1.4) | -0.60 (-1.27, 0.08) | 0.076 |
|  | - BPD | -0.32 (0.9) | -0.66 (1.2) | -0.33 (-1.10, 0.44) | 0.386 |
| **Gestational age,** weeks; mean (SD**)** | All EP | 26.7 (1.7) | 26.8 (1.6) | 0.01 (-0.7, 0.7) | 0.974 |
|  | - non BPD | 27.2 (1.7) | 27.3 (1.6) | 0.1 (-0.8, 1.1) | 0.788 |
|  | - BPD | 25.8 (1.5)* | 26.3 (1.4)** | 0.4 (-0.5, 1.4) | 0.381 |
| **Small for gestational age** **(SGA);** n (%) | All EP | 5 (14) | 20 (35) | 21 (2, 40) | 0.030 |
|  | - non BPD | 3 (13) | 12 (46) | 33 (7, 58) | 0.015 |
|  | - BPD | 2 (17) | 8 (26) | 9 (-19, 37) | 0.570 |
| **Postnatal days with oxygen treatment;** median (range) | All EP | 49 (2-180) | 65 (0-250) | 1.8 (1.0, 3.0) | 0.109 |
|  | - non BPD | 34 (2-70) | 44 (0-78) | 1.9 (-1.1, 3.4) | 0.254 |
|  | - BPD | 92 (61-180) | 79 (44-250) | -1.3 (-1.6, -1.2) | 0.080 |
| **Ventilator days;** median (range) | All EP | 4.0 (0-55) | 5.0 (0-24) | 1.2 (-1.4, 2.2) | 0.618 |
|  | - non BPD | 1.3 (0-40) | 2.5 (0-21) | 1.7 (-1.2, 3.2) | 0.212 |
|  | - BPD | 12.7 (2-55)*** | 8.0 (0-24)*** | -1.8 (-3.1, -1.1) | 0.011 |
| **Antenatal corticosteroids;** n (%) | All EP | 15/34 (44) | 46 (81) | 37 (17, 57) | ˂0.001 |
|  | - non BPD | 11 (48) | 21 (81) | 33 (6, 60) | 0.005 |
|  | - BPD | 4/11 (36) | 25 (81) | 45 (17, 73) | 0.012 |
| **Surfactant;** n (%) | All EP | 17 (49) | 49/56 (88) | 39 (20, 58) | ˂0.001 |
|  | - non BPD | 7 (30) | 20/25 (80) | 50 (22, 78) | 0.001 |
|  | - BPD | 10 (83) | 29 (94) | 11 (-8, 30) | 0.367 |
| **Postnatal corticosteroids;** n (%) | All EP | 10 (29) | 18 (32) | 3 (-16, 22) | 0.772 |
|  | - non BPD | 2 (9) | 1 (4) | 5 (-9, 19) | 0.549 |
|  | - BPD | 8 (67) | 17 (55)*** | 12 (-21, 45) | 0.510 |
| **Closing of PDA;** n (%) | All EP | 17 (49) | 13 (23) | 26 (6, 46) | 0.013 |
|  | - non BPD | 7 (30) | 3 (12) | 18 (-5, 41) | 0.122 |
|  | - BPD | 10 (83) | 10 (32) | 51 (18, 84) | 0.004 |
| **Maternal smoking in pregnancy;** n (%) | Control | 9 (26) | - | - | - |
|  | All EP | 13 (37) | 13/52 (25) | 12 (-8, 32) | 0.205 |
|  | - non BPD | 10 (43) | 5/24 (21) | 22 (-5, 49) | 0.111 |
|  | - BPD | 3 (25) | 8/28 (29) | 4 (-26, 34) | 0.957 |

Figures are means (SD), medians (ranges) or counts (%). **^a^** The p-value denotes differences between those born in 1991-92 and in 1999-2000. * P-values for group differences between EP non BPD vs. EP BPD within each cohort, * p˂0.05, **p˂0.01, ***p˂0.001.

**Table B**: Anthropometric data and respiratory symptoms at age 11 comparing children born preterm in 1991-92 (EP_1991-92_) and in 1999-2000 (EP_1999-2000_).

|  |  | **EP_1991-92_ cohort n=35** | **EP_1999-2000_ cohort n=57** | **Cohort Difference** | **p-value^a^** |
| --- | --- | --- | --- | --- | --- |
| **Age; years** | Control | 10.6 (0.4) | 11.7 (0.7) | 1.1 (0.8, 1.4) | ˂0.005 |
|  | All EP | 10.4 (0.4) | 11.4 (0.6) | 1.0 (0.8, 1.3) | ˂0.005 |
|  | - non BPD | 10.4 (0.5) | 11.4 (0.6) | 1.0 (0.7, 1.3) | ˂0.005 |
|  | - BPD | 10.4 (0.4) | 11.5 (0.6) | 1.1 (0.8, 1.4) | ˂0.005 |
| **Height;**z-score | Control | 0.02 (0.9) | 0.00 (1.1) | -0.02 (-0.46, 0.42) | 0.925 |
|  | All EP | -0.51 (1.2)* | -0.41 (1.0)* | 0.10 (-0.36, 0.57) | 0.654 |
|  | - non BPD | -0.52 (1.3) | -0.38 (1.0) | 0.14 (-0.51, 0.79) | 0.658 |
|  | - BPD | -0.49 (1.1) | -0.43 (1.1)) | 0.06 (-0.67, 0.79) | 0.868 |
| **Weight;** z-score | Control | 0.17 (1.0) | -0.14 (1.1) | -0.31 (-0.73, 0.12) | 0.153 |
|  | All EP | -0.48 (1.4)* | -0.33 (1.05) | 0.15 (-0.37, 0.66) | 0.570 |
|  | - non BPD | -0.40 (1.7) | -0.43 (1.0) | -0.02 (-0.80, 0.76) | 0.956 |
|  | - BPD | -0.61 (0.8) | -0.25 (1.1)) | 0.37 (-0.34, 1.08) | 0.303 |
| **BMI;** z-score | Control | 0.25 (0.9) | -0.22 (1.0) | -0.46 (-0.89, 0.04) | 0.033 |
|  | All EP | -0.28 (1.4) | -0.16 (1.0) | 0.12 (-0.39, 0.62) | 0.644 |
|  | - non BPD | -0.18 (1.6) | -0.35 (1.1) | -0.17 (-0.93, 0.60) | 0.660 |
|  | - BPD | -0.46 (0.9) | 0.00 (1.0) | 0.46 (-0.19, 1.11) | 0.178 |
| **Asthma ever** | Control | 3 (9) | 5 (9) | 0 (-12, 12) | 0.933 |
|  | All EP | 12 (34)* | 15 (26)* | 8 (-11, 27) | 0.427 |
|  | - non BPD | 6 (26) | 5 (19) | 7 (-16, 30) | 0.587 |
|  | - BPD | 6 (50) | 10 (32) | 18 (-14, 50) | 0.309 |
| **Asthma medication last 12 months** | Control | 1 (3) | 3 (6) | 3 (-6, 12) | 0.838 |
|  | All EP | 5 (14) | 4 (7) | 56 (35, 77) | 0.075 |
|  | - non BPD | 1 (4) | 3 (12) | 8 (-7, 23) | 0.775 |
|  | - BPD | 4 (33) | 1 (3) | 3 (13, 47) | 0.006 |
| **Wheeze last 12 months** | Control | 2 (6) | 5 (9) | 3 (-8, 14) | 0.587 |
|  | All EP | 11 (31)* | 8 (14) | 17 (0-34) | 0.055 |
|  | - non BPD | 6 (26) | 4 (15) | 11 (-12, 34) | 0.383 |
|  | - BPD | 5 (42) | 4 (13) | 29 (18, 56) | 0.060 |
| **Atopy** | Control | 8 (23) | 20/50 (40) | 17 (-14, 47) | 0.105 |
|  | All EP | 9 (26) | 12/55 (22) | 4 (-14, 22) | 0.675 |
|  | - non BPD | 8 (35) | 6/25 (24) | 11 (-15, 37) | 0.435 |
|  | - BPD | 1 (8) | 6/30 (20) | 12 (-13, 37) | 0.415 |

Figures are means (SD), medians (ranges) or counts (%). **^a^** The p-value denotes differences between those born on 1991-92 and in 1999-2000. * P-values for group differences between term controls vs. all EP within each cohort, * p˂0.05. Atopy registered as minimum one positive SPT (skin prick test) or IgE test in the EP_1991-92_ cohort and as minimum one positive SPT in the EP_1999-2000_ cohort.

**Hospital admissions and respiratory symptoms in EP_1999-2000_**

Hospital admissions during the 5 years prior to inclusion were more common in the EP than the term-born group (16/55 vs. 7/53; p=0.044), more so for those at GA ≤25 weeks than GA 26-27 weeks (5/9 vs. 6/35, p=0.030), with no influence from BPD. Respiratory causes were given as reason for admittance in 2 EP and 1 term-born subject. More EP than term-born subjects had ever been diagnosed with asthma (15 vs. 5; OR 3.5; 95%CI 1.2, 10.4), but current asthma did not differ (6 vs. 4; OR 1.5, 95%CI 0.4, 5.5). Current asthma was present in 3/10, 2/36 and 1/11 of those born at GA ≤ 25 weeks, GA 26-27 weeks and GA ≥ 28 weeks, respectively; i.e. significantly more in the most immature group vs. the GA 26-27 weeks group (OR 7.29; 95%CI 1.02, 52.01). Respiratory symptoms were rare and similarly distributed between EP and term-born; i.e. respectively 8 vs. 5 (OR 1.60, 95%CI 0.49, 5.24) had wheeze the last 12 months, 10 vs. 3 (OR 3.79; 95%CI 0.98, 14.65) had used asthma medication after 5 years of age, and 5 vs. 2 (OR 2.50; 95%CI 0.46, 13.47) had lower respiratory tract infections treated by antibiotics after 5 years of age.

**Lung function**

Flow-volume loops were satisfactorily obtained from all participants, static lung volumes were successfully measured in 53/57 EP and 47/54 term-born subjects, bronchial hyperresponsiveness to methacholine (DRS) was successfully assessed in 55/57 EP and 50/54 term-born subjects, reversibility to salbutamol was successfully assessed in 56/57 EP and 53/54 term-born subjects, Fe_NO_ was successfully measured in 49/57 EP and 48/54 term-born subjects, with alveolar NO obtained from 45/57 and 43/54, respectively, missing data mainly due to equipment failure the first months of the study, and DLCO was successfully measured in in 43/57 EP and 45/54 term-born subjects.

**Table C:** Comparison of two cohorts of subjects born preterm eight years apart, in 1991-92 (EP_1991-92_) and in 1999-2000 (EP_1999-2000_).

|  |  | **EP_1991-92_** | | | **EP_1999-2000_** | | |
| --- | --- | --- | --- | --- | --- | --- | --- |
|  |  | Control | EP non BPD | EP BPD | Control | EP non BPD | EP BPD |
| FEV_1_ | z | -0.05 (-0.35, 0.25) | -0.56 (-0.91, -0.20) | -1.52 (-2.06, -0.97) | -0.31 (-0.57, -0.04) | -0.55 (-0.88, -0.22) | -0.73 (-1.10, -0.37) |
| FVC | z | -0.05 (-0.35, 0.25) | -0.57 (-0.90, -0.23) | -1.54 (-2.08, -1.00) | -0.16 (-0.42, 0.09) | -0.17 (-0.48, 0.13) | -0.17 (-0.54, 0.20) |
| FEV_1_/FVC | z | 0.10 (-0.21, 0.42) | 0.02 (-0.42, 0.46) | -1.40 (-2.01, -0.80) | -0.30 (-0.54, -0.05) | -0.69 (-1.06, -0.31) | -0.90 (-1.29, -0.52) |
| FEF_25-75_ | z | -0.22 (-0.54, 0.10) | -0.74 (-1.10, -0.39) | -1.92 (-2.49, -1.36) | -0.53 (-0.79, -0.27) | -1.04 (-1.40, -0.68) | -1.22 (-1.58, -0.87) |
| DRS | G.mean* | 3.0 (1.7, 5.2) | 10.8 (5.3, 22.0) | 33.4 (10.6, 105.0) | 3.47 (2.19, 5.50) | 11.48 (5.75, 23.44) | 14.79 (7.24, 29.51) |
| TLC | z | 0.18 (-0.15, 0.52) | -0.06 (-0.37, 0.26) | 0.37 (-0.12, 0.86) | 0.45 (0.13, 0.76) | 0.17 (-0.11, 0.46) | 0.41 (0.00, 0.82) |
| FRC | z | -0.20 (-0.61, 0.20) | -0.71 (-1.18, -0.25) | -0.24 (-0.50, 0.98) | -0.34 (-0.74, 0.06) | -0.26 (-0.67, 0.15) | 0.36 (-0.16, 0.89) |
| RV | z | -0.31 (-0.66, 0.04) | -0.48 (-0.90, -0.05) | 0.49 (-0.11, 1.08) | 0.27 (-0.09, 0.63) | 0.05 (-0,40, 0.51) | -0.04 (-0.47, 0.38) |
| RV/TLC | % | 24.3 (22.5, 26.1) | 24.8 (22.0, 27.7) | 30.1 (26.2, 33.9) | 26.2 (24.4, 27.9) | 26.9 (24.0, 29.8) | 25.2 (22.4, 27.9) |
| Raw | z | 0.55 (0.31, 0.79) | 0.73 (0.46, 1.01) | 1.31 (-0.54, 3.15) | 0.68 (0.51, 0.85) | 0.96 (0.72, 1.21) | 1.45 (0.61, 2.29) |

Figures are observed means (95 % confidence intervals) for two cohorts of EP born subjects. * For DRS (geometric means), a difference between two values is negative if less than 1 and positive if larger than 1.

T**able D:** Perinatal data for 57 preterm (EP) born participants born in 1999-2000, split by the presence or absence of bronchopulmonary dysplasia (BPD) and by gestational age (GA) categories.

|  | **EP non BPD** N=26 | **EP BPD** N=31 | **GA ≤25 weeks** N=10 | **GA 26-27 weeks** N=36 | **GA ≥28 weeks** N=11 |
| --- | --- | --- | --- | --- | --- |
| **Gestational age**; weeks | 27.3 (1,6) | 26.3 (1,4)** | 24.4 (0.5) | 26.7 (0.5) | 29.2 (1.0) |
| **Birthweight**; gram | 872.5 (200) | 830.6 (151) | 741.2 (107) | 904.1 (177) | 770.1 (146) |
| **Birthweight**; z | -0.97 (1.4) | -0.67 (1.2) | 0.09 (0.9) | -0.54 (1.0) | -2.46 (0.7)*** |
| **SGA**; n | 12 (46) | 8 (26) | 1 (10) | 8 (22) | 11 (100)*** |
| **BPD**, n | - | - | 8 (80) | 20 (56) | 3 (27)*** |
| **Antenatal steroids**; n | 21 (81) | 25 (81) | 8 (80) | 28/34 (82) | 10 (91) |
| **Surfactant**; n | 20/25 (80) | 29 (94) | 10 (100) | 32/35 (91) | 7 (64)* |
| **Days on ventilator** (range) | 3 (0-21) | 8 (0-24)*** | 12 (4-24) | 4 (0-17) | 3 (0-9)** |
| **Days with CPAP** (range) | 20 (0-53) | 36 (4-81)*** | 46 (28-72) | 28 (2-81) | 4 (0-50)*** |
| **Days with oxygen** | 44 (22) | 79 (35)*** | 80 (26) | 66 (38) | 30 (36)* |
| **Systemic steroids**; n | 1 (4) | 17 (55)*** | 8 (80) | 10 (28) | 0*** |
| **PDA**; n | 3 (12) | 10 (32) | 4 (40) | 8 (22) | 1 (9) |
| **Intracerebral pathology**; n | 6 (23) | 8 (26) | 5 (50) | 7 (19) | 2 (18) |
| **ROP**; n | 1/21 (5) | 4/28 (14) | 4 (40) | 0 | 1/10 (10)*** |
| **NEC**; n | 0 | 1/26 (4) | 0 | 1 (3) | 0 |
| **Maternal smoking**; n | 5/24 (21) | 8/28 (29) | 1/9 (11) | 7/35 (20) | 2/8 (25) |

Figures are means (SD), medians (range) or counts (%). * refers to p-values; *=p˂0.05, **=p˂0.01, ***=p˂0.001 for the differences between groups based on BPD or GA. Abbreviations: SGA = small for gestational age, i.e. birthweight below 10^th^ percentile; antenatal corticosteroids registered if administered in the last week pre labor, surfactant registered if administered in the rescue room or in the neonatal intensive care unit (NICU); CPAP = continuous positive airway pressure; PDA = persistent ductus arteriosus, here registered if treated by medical (indometacine) or surgical intervention; intracerebral pathology registered if intracerebral bleeding, distended side ventricles or periventricular leukomalacia noted on ultrasound scan assessments in the NICU; ROP = retinopathy of prematurity, here registered if treated by cryosurgery; NEC = necrotizing enterocolitis, here registered if treated surgically; maternal smoking registered as daily or occasional smoking during pregnancy.

# References

1. Juliusson PB, Roelants M, Nordal E, Furevik L, Eide GE, Moster D, et al. Growth references for 0-19 year-old Norwegian children for length/height, weight, body mass index and head circumference. Annals of human biology. 2013. Epub 2013/02/19. doi: 10.3109/03014460.2012.759276. PubMed PMID: 23414181.

2. Quanjer PH, Tammeling GJ, Cotes JE, Pedersen OF, Peslin R, Yernault JC. Lung volumes and forced ventilatory flows. Report Working Party Standardization of Lung Function Tests, European Community for Steel and Coal. Official Statement of the European Respiratory Society. The European respiratory journal Supplement. 1993;16:5-40. Epub 1993/03/01. PubMed PMID: 8499054.

3. Cotes JE, Chinn DJ, Quanjer PH, Roca J, Yernault JC. Standardization of the measurement of transfer factor (diffusing capacity). Report Working Party Standardization of Lung Function Tests, European Community for Steel and Coal. Official Statement of the European Respiratory Society. The European respiratory journal Supplement. 1993;16:41-52. Epub 1993/03/01. PubMed PMID: 8499053.

4. Macintyre N, Crapo RO, Viegi G, Johnson DC, van der Grinten CP, Brusasco V, et al. Standardisation of the single-breath determination of carbon monoxide uptake in the lung. Eur Respir J. 2005;26(4):720-35. Epub 2005/10/06. doi: 10.1183/09031936.05.00034905. PubMed PMID: 16204605.

5. Wanger J, Clausen JL, Coates A, Pedersen OF, Brusasco V, Burgos F, et al. Standardisation of the measurement of lung volumes. Eur Respir J. 2005;26(3):511-22. Epub 2005/09/02. doi: 10.1183/09031936.05.00035005. PubMed PMID: 16135736.

6. Miller MR, Hankinson J, Brusasco V, Burgos F, Casaburi R, Coates A, et al. Standardisation of spirometry. Eur Respir J. 2005;26(2):319-38. Epub 2005/08/02. doi: 10.1183/09031936.05.00034805. PubMed PMID: 16055882.

7. Quanjer PH, Stanojevic S, Cole TJ, Baur X, Hall GL, Culver BH, et al. Multi-ethnic reference values for spirometry for the 3-95-yr age range: the global lung function 2012 equations. Eur Respir J. 2012;40(6):1324-43. Epub 2012/06/30. doi: 10.1183/09031936.00080312. PubMed PMID: 22743675.

8. Rosenthal M, Cramer D, Bain SH, Denison D, Bush A, Warner JO. Lung function in white children aged 4 to 19 years: II--Single breath analysis and plethysmography. Thorax. 1993;48(8):803-8. Epub 1993/08/01. PubMed PMID: 8211869; PubMed Central PMCID: PMC464705.

9. American Thoracic S, European Respiratory S. ATS/ERS recommendations for standardized procedures for the online and offline measurement of exhaled lower respiratory nitric oxide and nasal nitric oxide, 2005. Am J Respir Crit Care Med. 2005;171(8):912-30. Epub 2005/04/09. doi: 10.1164/rccm.200406-710ST. PubMed PMID: 15817806.

10. Hogman M. Extended NO analysis in health and disease. Journal of breath research. 2012;6(4):047103. Epub 2012/06/09. doi: 10.1088/1752-7155/6/4/047103. PubMed PMID: 22677778.

11. Sepponen A, Lehtimaki L, Huhtala H, Kaila M, Kankaanranta H, Moilanen E. Alveolar and bronchial nitric oxide output in healthy children. Pediatr Pulmonol. 2008;43(12):1242-8. Epub 2008/11/15. doi: 10.1002/ppul.20953. PubMed PMID: 19009623.

12. Nieminen MM, Lahdensuo A, Kellomaeki L, Karvonen J, Muittari A. Methacholine bronchial challenge using a dosimeter with controlled tidal breathing. Thorax. 1988;43(11):896-900. Epub 1988/11/01. PubMed PMID: 3065974; PubMed Central PMCID: PMC461551.

13. Crapo RO, Casaburi R, Coates AL, Enright PL, Hankinson JL, Irvin CG, et al. Guidelines for methacholine and exercise challenge testing-1999. This official statement of the American Thoracic Society was adopted by the ATS Board of Directors, July 1999. Am J Respir Crit Care Med. 2000;161(1):309-29. Epub 2000/01/05. doi: 10.1164/ajrccm.161.1.ats11-99. PubMed PMID: 10619836.

14. O'Connor G, Sparrow D, Taylor D, Segal M, Weiss S. Analysis of dose-response curves to methacholine. An approach suitable for population studies. The American review of respiratory disease. 1987;136(6):1412-7. Epub 1987/12/01. doi: 10.1164/ajrccm/136.6.1412. PubMed PMID: 3318599.

15. Pellegrino R, Viegi G, Brusasco V, Crapo RO, Burgos F, Casaburi R, et al. Interpretative strategies for lung function tests. Eur Respir J. 2005;26(5):948-68. Epub 2005/11/03. doi: 10.1183/09031936.05.00035205. PubMed PMID: 16264058.

16. Position paper: Allergen standardization and skin tests. The European Academy of Allergology and Clinical Immunology. Allergy. 1993;48(14 Suppl):48-82. Epub 1993/01/01. PubMed PMID: 8342740.
